# Task Specific Tremor in Parkinson’s Disease Responds to Apomorphine

**DOI:** 10.5334/tohm.764

**Published:** 2023-06-02

**Authors:** William G. Ondo, Vindhya Koneru, Chia Arif

**Affiliations:** 1 Houston Methodist Neurological Institute, Houston Methodist Hospital, Houston, Texas, Weill Cornel Medical School, US; 2 Houston Methodist Neurological Institute, Houston Methodist West Hospital, Houston, US; 3 St. Mary Medical Center-Langhorne, PA, US

**Keywords:** Task specific tremor, writing tremor, Parkinson’s disease, apomorphine, DaTscan

## Abstract

**Background:**

Task specific tremor (TST) is a poorly understood entity without any standard treatments, that may subsequently develop tremor during additional tasks, later develop postural/kinetic tremor (essential tremor criteria), and later develop Parkinson’s disease. The pathophysiology is not understood as it has features of tremor, dystonia, and parkinsonism.

**Objectives:**

To assess response of TST to apomorphine and thus infer pathophysiology.

**Methods:**

We administered sublingual apomorphine to 8 patients diagnosed with Parkinson’s disease based on clinical criteria and dopamine imaging, who all initially presented with TST and later presented other parkinsonian signs and dopamine imaging deficits.

**Results:**

Apomorphine improved TST, which was refractory to oral levodopa and other tremor therapies, in 6/8 subjects.

**Discussion:**

These results offer a treatment option for TST, which is usually refractory to other pharmacologic treatments, in patients with other parkinsonian features, and infers a dopaminergic pathophysiology of TST.

Task specific tremor (TST) is a poorly understood condition manifest by a high amplitude focal tremor seen when performing a specific task, most commonly writing [1,2,3]. The pathology of TST is debated, with some arguing it is more similar to dystonia than tremor [4]. However, TST is phenomenologically distinct from dystonic tremor, which is typically a lower amplitude, often jerky tremor seen in conjunction with focal hand dystonia or with distant dystonia, usually cervical [5,6]. TST is not codified as essential tremor (ET) [7], although it may later evolve such that the tremor occurs with additional tasks, or eventually demonstrates a postural tremor, sometimes with a crescendo component, and then could be diagnosed as ET [1]. TST patients may also develop a true rest tremor, and patients may develop Parkinson’s disease (PD) based on clinical criteria and Ioflupane I^123^ (DaTscan^TM^) imaging [8]. Purely unilateral action tremor, which sometimes is task specific, is also reported to slowly evolve into PD [9].

There are no published treatment trials for TST. It appears less responsive to ethanol and traditional tremor medications (propranolol, primidone, topiramate) compared to ET, but formal comparisons are lacking [1,2]. Botulinum toxin (BoNT), and surgical interventions are often used. There is a case report of TST improving with L-dopa [10].

We report a series of patients presenting with, and continuing to have refractory TST, who later developed Parkinson’s disease based on clinical criteria including rest tremor, corroborated by dopamine imaging. We evaluated the impact of sublingual apomorphine (SL Apo) on their TST.

## Methods

Patients with TST/PD were seen at the Methodist Hospital Movement Disorders Clinic. An initial 10 mg dose of sublingual apomorphine (SL Apo) was administered in clinic, as per our standard of care, and patients were allowed to titrate at home if needed. After signing informed consent, patients were videotaped before and after an initial dose, and in some cases at a subsequent visit after SL Apo dose optimization.

## Results

All subjects initially reported an action tremor with one specific task, although some have progressed to have action tremor in other tasks by our initial evaluation [Table 1]. The TSTs were are all high amplitude, and in individual subjects had a fairly homogeneous frequency, anatomic involvement, and direction, without dystonic posturing.

We gave SL Apo to 8 patients whose PD signs were preceded, or in one case approximately concurrent, with TST: handwriting (6), holding a cup (1), jaw drinking tremor (1) [Table 1]. DaTscan^TM^ confirmed variable dopamine loss in all cases. No other attempted pharmacotherapy satisfactorily improved TST [Table 1], although L-dopa improved other PD symptoms, including rest tremor, and caused dyskinesia in the 2 subjects with more severe PD features. L-dopa mildly improved TST in one case but showed no benefit in the other seven.

**Table 1:** B-block = Beta-blocker (nadolol or propranolol), BoNT = botulinum toxin-A, L-dopa = levodopa and maximum individual dose tried, rop = ropinirole, trihex = trihexyphenidyl, Li+ = Lithium.


	Sex	Age (y)	TST phenotype	Age onset TST	Age onset PD	DaTscan Abnormal	Comment	Previous Inadequate Treatments for TST	Apomorphine Response/Disposition/Adverse Events

1	M	70	Writing, later other tasks	59	59	Bilat Marked Loss R > L	R hand writing tremor, L hand rest tremor	L-dopa 250mg, pramipexole	Good/ Stopped- low benefit to AE ratio/ Nausea, subjective hypertension

2	M	63	Writing	48	67	Bilat Moderate Loss	+Fam Hx ET, Marked PD signs and dyskinesia	Zonisamide*, trihex, rop, L-dopa* 200mg, amantadine	Very good/ Cont 20 mg until STN DBS^/ None

3	M	76	Writing, later other tasks	65	72	Bilat Mild Loss	Mild PD, Orthostatic hypotension	L-dopa 200mg, BoNT*, zonisamide, primidone	None (up to 10 mg)/ Stopped after single 10mg dose/ Severe vomiting and syncope

4	M	78	Writing, later spoon left-handed#	72	76	Mild Unilat R Side Loss	OCD, Startle response, Mild PD signs	L-dopa 250mg	Very Good/ Cont 20 mg/ None

5	M	60	Writing, later using utensils	45	58	Mod R > L Post. Loss	Mild PD signs	L-dopa 200mg, B-block	Moderate/ Stopped- low benefit to AE ratio/ Nausea, sleepiness, foggy

6	M	73	Writing, later using utensils	50	70	R > L Mod Loss	Re-emergent tremor, Mild PD signs	BoNT*, L-dopa 200mg, prim, topiramate, B-block, amantadine	None (up to 25 mg)/ Stopped/ Nausea

7	M	69	Holding cup, then writing	64	68	Mild Unilat Left Put Loss	Takes Li+ for bipolar, Mild PD signs	Amantadine, L-dopa 500mg	Excellent/ Takes occasionally/ None

8	F	87	Jaw drinking tremor	74	82	L > R Mod Loss	Marked PD signs and dyskinesia	L-dopa 500mg, istradefylline, amantadine, BoNT	Excellent/ Continues 20 mg dose 1–2/day/ None


*Partial response to treatment, otherwise no response. #All other subjects were Right hand dominant. ^STN DBS also markedly improved all large amplitude action tremor.

SL Apo improved the TST markedly (4), moderately (2), none (1), or was not tolerated (1) [Videos 1–3]. Although effective, SL Apo was eventually discontinued in 2 patients who responded, secondary to lack of subjective disability from their TST, combined with moderate adverse events, and in 1 patient because of successful treatment of TST with sub-thalamic nucleus deep brain stimulation.

**Videos 1–3:** **Patient 1 (top), Patient #2 (middle), and Patient #7 (bottom).** Writing tremor and selected other action tremors in 3 of the subjects (#1, #2, #7) before apomorphine (with or without L-dopa) and shortly after sub-lingual apomorphine.

## Discussion

The TST in this group of patients often responded to apomorphine but was refractory to other dopaminergics and traditional tremor treatments. Other PD signs, including rest tremor did respond to both L-dopa and apomorphine, as would be expected.

The mechanism by which apomorphine improved TST is not known. Overall response of L-dopa and apomorphine is felt to be comparable in advanced PD, and apomorphine’s greater clinical potency, compared to other dopamine agonist, is attributed to its rapid penetration across the blood brain barrier rather than higher affinity for dopamine receptors. Compared to dopamine, apomorphine has relatively similar dopamine receptor subtype affinities, except a greater affinity for D4 receptors and relatively greater D2:D3 affinity ratio. Whether very high doses of L-dopa could have improved TST is not known but two of the patients tried 500 mg L-dopa doses without any TST improvement. Non-dopaminergic affinities for apomorphine are fairly low, but it is a modest 5HT_2(a-c)_ agonist and a_2a-c_ adrenergic antagonist. Since the pathophysiology of TST is largely unknown, attributing other mechanisms would be highly speculative.

This is a retrospective review and suffers limitations inherent to that study type. There was no prospective protocol, so data collection was inconsistent. For example, we did not capture complete UPDRS nor TETRAS data, and there is no scale specifically for TST, so efficacy was based solely on global impressions of improvement in their TST with individual apomorphine doses in clinic. We did not track home use. Some patients were seen when they only had TST without any other action or rest tremor phenotype but in others this diagnosis was based on history from the patient and outside medical records. Although all patients had very prominent action tremor, the TST was no longer “specific” in some patients at the time of apomorphine intervention. Clinically, the short duration of efficacy per dose, and relatively high side effect profile, makes the treatment practical for only select cases. Some patients with TST also discontinued apomorphine because their TST component was not problematic enough to add another permanent medication.

We have not tried “off label” apomorphine on TST subjects without any clinical or imaging evidence of parkinsonism. However, TST response to apomorphine further suggests TST is related to dopaminergic dysfunction and strengthens its association with PD.
